# Changes to radiotherapy utilisation in Western NSW after the opening of a local service

**DOI:** 10.1002/jmrs.204

**Published:** 2017-02-03

**Authors:** Sally M. Butler

**Affiliations:** ^1^ Central West Cancer Service, Orange Health Service Orange New South Wales Australia

**Keywords:** Health services, patient access, radiation oncology, rural, travel distance

## Abstract

**Introduction:**

In 2011, the first radiotherapy centre in Western NSW Local Health District (WNSWLHD) was opened in the city of Orange. Prior to this, patients travelled outside the health service, primarily to Sydney, to receive treatment. The aim of this study was to investigate if the establishment of the new rural radiotherapy service has changed the demographic profile, cancer type, treatment intent and number of patients treated.

**Methods:**

Data were collected on WNSWLHD patients, 17 years of age and above, who received radiotherapy in either 2010 or 2012 in New South Wales (NSW) or Australian Capital Territory (ACT). The age, gender, treatment intent, cancer type and residential town were recorded.

**Results:**

The number of patients who accessed radiation increased from 573 to 667 between 2010 and 2012. The corresponding radiotherapy utilisation (RTU) rates were 29.3% in 2010 and 33.4% in 2012, an improvement of 4.1% (*P* = 0.01, 95% CI 1–7%). Patients travelled 128.5 km less for treatment in 2012 than in 2010 (338.7 km vs. 210.2 km, CI 111–145.5 km, *P* > 0.0001). All regions had an improvement in the RTU rates apart from the Remote region which decreased by 9% (31–20% in 2012). The number of palliative treatments increased significantly only within the Orange region. The number of male patients for treatments also significantly increased as there were 81 additional treatments (292 vs. 373) as did patients with a respiratory cancer (66 vs. 97).

**Conclusions:**

A new radiotherapy service in a sparsely populated health district significantly changed the pattern of radiotherapy use for those who lived only in the Orange region. Treatment capacity at the Orange radiotherapy centre has doubled with the opening of a second linear accelerator since this study was conducted. Thus, a follow‐up study is recommended to ascertain if radiotherapy rates remain low in the regions beyond Orange.

## Introduction

In theory, it is estimated that 48.3% of all cancer patients should receive radiotherapy during the course of their disease.^1^ However, actual radiotherapy rates have been found to be much lower ranging from 26% in the United States of America (2010–2012), 33% in Canada (2008) and 26% in New South Wales (NSW) and the Australian Capital Territory (ACT) in 2004–2006.^2^, ^3^, ^4^ Factors contributing to the difference between the optimal and actual radiotherapy utilisation (RTU) rates include accessibility to services, referral by service providers or patient preference.^5^, ^6^, ^7^

**Figure 1:**
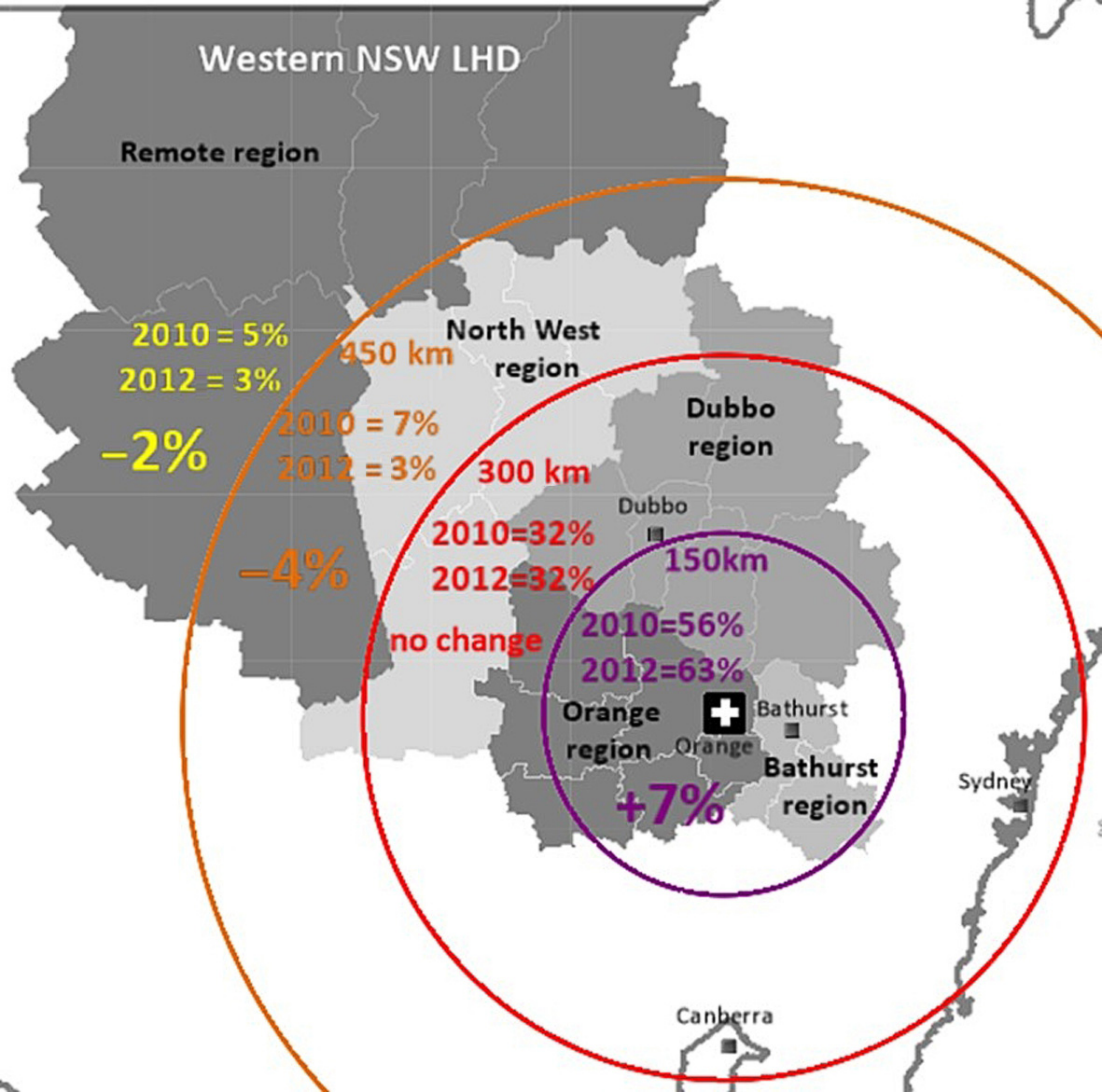
Distances (km) from participant's residential location to Orange; km, kilometres.

Western New South Wales Local Health District (WNSWLHD) is the second most sparsely populated Local Health District (LHD) in NSW. It covers 31% of NSW and has a population density of one person per square kilometre. Figure 1 shows a map of the regions within WNSWLHD. Around one‐third of the LHD are resident in the Orange region; one‐third in the Dubbo region; 17% in the Bathurst region; 6% in the North West region and 6% in the Remote region. Dubbo, Bathurst and Orange all have a similar level of specialist availability.^8^

Prior to the WNSWLHD radiation service, Dubbo, Bathurst and Orange were serviced by outreach clinics. If radiotherapy was required the visiting radiation oncologist would primarily refer the cancer patient to a radiation centre in Sydney. In May 2011, a radiation centre was built in Orange and the outreach clinics at Orange and Bathurst discontinued. The outreach clinic in Dubbo was continued due to limited capacity at the new radiation centre in Orange.^9^ This situation provided a unique opportunity to analyse two different health service models in two similar rural areas of population size, specialists and services.

In other rural areas of Australia where radiotherapy centres have opened, the results have been positive. In Victoria, the single machine unit (SMU) trial increased radiotherapy services to three rural regions. The utilisation rates increased by 12% for rural people compared to 6% of metropolitan Melbourne between 2001 and 2003/2004. It also showed that in Victoria the overall utilisation rate increased from 37.4% to 39.0%, and in rural areas, the utilisation rate increased by 8%.^10^

The difference between this study and the SMU trial is that the three rural health regions in the SMU trial had a population density five times larger than WNSWLHD. More recently, there has been data published on the changes to travel distance for prostate and breast cancer patients in North Queensland. The study found that an additional radiation centre in North Queensland decreased the average distance travelled. The North Queensland study did not consider differences between palliative and curative treatments, all tumour groups or residents who had treatment at facilities outside North Queensland.^11^

Our study aimed to examine how radiotherapy treatment patterns have changed, to what extent, and for which population groups. It also determined whether or not the changes have been widespread or limited to the region surrounding the new service, located in the city of Orange. Therefore, the findings of our study build upon current radiotherapy and rural health service planning knowledge.

All participants in this study lived in a rural area. Rural was defined as a residential town with an Accessibility Remoteness Index of Australia (ARIA) score greater than 0.2, 100 km from a capital city and with a local government area population of less than 50,000 people.^12^

## Methods

This is a repeat cross‐sectional study. Data was collected from WNSWLHD cancer patients who had both curative (radical) and palliative radiotherapy in the year prior to the new service (2010) and compared with WNSWLHD cancer patients treated in the first year that the new service was at full capacity (2012).

The selection criteria were:

- cancer patients 17 years of age and over;
- a residential address within WNSWLHD;
- received radiotherapy in the calendar years of 2010 or 2012;
- treated with a megavoltage course in NSW or ACT; and
- diagnosed with a NSW Health notifiable cancer.^13^

Data was collected directly from every radiotherapy centre in NSW and ACT. This equated to 22 in total, 21 centres within NSW (15 public + 6 private) and one public centre is in the ACT. This approach reduced selection bias as it is estimated that 99% of WNSWLHD patients were referred to radiotherapy centres in NSW and ACT in 2010 and 2012.^14^, ^15^

Cancer patients who had radiotherapy in 2011 were excluded because the WNSWLHD service took until the end of 2011 to reach full capacity.^9^ Orthovoltage, brachytherapy and complex treatments were also excluded because these are not available at all radiotherapy centres. It is estimated that in both 2010 and 2012 megavoltage courses accounted for 91% of all radiotherapy courses delivered in NSW and ACT.^14^, ^15^ Therefore, exclusion of orthovoltage, brachytherapy and complex treatments would have had minimal effect on the RTU rates found in our study.

### Data extraction

Data managers from each centre extracted the required information, de‐identified the patient data then transferred it to the coordinating principal investigator. Received information contained participants’; residential town and postcode; cancer diagnosis or International Classification of Diseases code; age at treatment; treatment intent; place of treatment; gender; and year treated with radiotherapy.

### Data analysis

The radiotherapy utilisation rate is defined as the number of new radiotherapy courses divided by the number of new cancer cases in the same place and time. As no patient identifiable details were collected, it was not known if the same patient was treated more than once. To find the number of new radiotherapy courses, a retreatment rate of 26.4% was subtracted from the total number of treatments. This retreatment rate was based on the NSW Radiotherapy Management Information System (RMIS) retreatment rate in 2010 and 2012.^14^, ^15^

The number of new cancer cases was calculated using 2008 NSW Cancer Institute data and adjusting it to the 2010 and 2012 populations.^16^, ^17^, ^18^ Five‐year cancer prevalence was mainly used as the denominator for chi‐square analysis. Prevalence was calculated using the same method to calculate cancer incidence. Where prevalence could not be used, such as for treatment intent, the proportion of one variable to another was compared, that is palliative to curative treatment.

Geographical regions within WNSWLHD were analysed as they represent areas of commonality such as remoteness, specialist availability and treatment services. They were drawn from the WNSWLHD Health Needs Assessment, which defined areas by combining Local Government Areas with similar ARIA and SEIFA (Socio‐Economic Indexes for Areas) scores.^8^ Google maps were used to measure the distance in kilometres participants travelled.

Independent samples *T*‐tests were used for continuous variables, chi‐square for categorical variables and univariate logistic regression to compare significance between regions. A *P*‐value of less than 0.05 was considered significant. SPSS version 22 was the statistical package used to analyse results.

### Ethics approval

Ethics approval was obtained from the following Human Research Ethics Committees: Greater Western (LNR/12/GWAHS/92); ACT Health (ETHLR.12.286); Riverina Cancer Care Centre; Macquarie University (5201300437); and ethically ratified by St Vincent's Hospital (LNR/12/GWAHS/92: Ethically Ratified).

## Results

**Figure 2:**
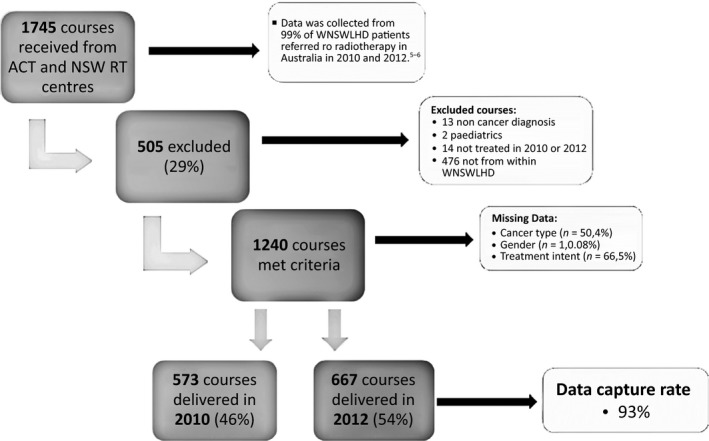
Progression of records for data analysis.

The progression of records for data analysis is shown in Figure 2. Of the 1745 courses received, 1240 met the selection criteria. The main reason for exclusion was that the recorded residential address was not within WNSWLHD. Eighty‐seven (7%) of the courses that met the selection criteria were missing either, cancer type, gender and/or treatment intent. The estimated values for treatment intent were analysed to check if the missing data altered the results. The *P*‐value was very similar to the *P*‐value obtained from the original data (*P* = 0.11 vs. *P* = 0.15), showing that the missing data did not affect results.

In total, there were 94 additional radiotherapy treatments between 2010 and 2012 (573 vs. 667), which represents a 14% increase over the study period. The RTU rate was found to be 29.3% in 2010 and 33.4% in 2012, an increase of 4.1% (*P* = 0.01 95% CI 1–7%).

### Geographical location

Figure 1 is a map showing 2010 and 2012 proportional differences between the 150 km distances from participant's residential town to the new service. Participants who lived within 150 km of the new service had the highest proportional increase (7%). Between 151 and 300 km, there was no change to the proportion treated and past 300 km the proportion treated decreased during the study period. This shows a trajectory of worsening outcomes as distance from the new service increased.

The average distance (km) from each participant's residential town to the radiotherapy centre at which they were treated decreased by 128.5 km in 2012 (338.7 km vs. 210.2 km, CI 111–145.5 km, *P* > 0.0001). This decrease was significant, however, it should be noted that with the presence of a local service in 2012, the average distance a WNSWLHD patient travelled for treatment remained high (210.2 km).

Table 1 shows that the number of radiotherapy treatments within the Orange region increased significantly over the study period (*P* = 0.001), with minimal increase in the Bathurst, Dubbo and North West regions. The number of radiotherapy treatments in the Remote region decreased by 36%, which was not statistically significant when prevalence is used as the denominator (33 vs. 21, *P* = 0.08).

**Table 1:** Number of radiation treatments by region, treatment intent, gender, age, and tumour group

Variable	Grouping	2010, *n* (%)	2012, *n* (%)	OR (95% CI)	*P* value^a^
Region	Bathurst	86 (15%)	102 (15%)	1.2 (0.9–1.6)	0.28
	Orange	219 (38%)	296 (44%)	1.4 (1.1–1.7)	0.001
	Dubbo	195 (34%)	206 (31%)	1.0 (0.8–1.3)	0.69
	North West	40 (7%)	42 (6%)	1.0 (0.7–1.6)	0.90
	Remote	33 (6%)	21 (3%)	0.6 (0.3–1.1)	0.08
Treatment intent	Palliative	175 (31%)	254 (38%)	1.2 (0.9–1.5)	0.15
	Curative	336 (59%)	409 (61%)		
Gender	Male	292 (51%)	373 (56%)	1.3 (1.1–1.5)	0.002
	Female	280 (49%)	294 (44%)	1.0 (0.9–1.2)	0.75
Age (years)	17–49	66 (12%)	58 (9%)	0.9 (0.6–1.3)	0.46
	50–64	205 (36%)	224 (34%)	1.1 (0.9–1.3)	0.48
	65–79	237 (41%)	294 (44%)	1.2 (1.0–1.4)	0.08
	80+	65 (11%)	91 (14%)	1.4 (1.0–1.9)	0.07
Tumour group	Breast	136 (24%)	146 (22%)	1.1 (0.8–1.4)	0.64
	Urogenital	109 (19%)	139 (21%)	1.3 (1.0–1.7)	0.07
	*>(Prostate)* b	*92 (16%)*	*123 (18%)*		
	Respiratory	66 (12%)	97 (15%)	1.8 (1.2–2.7)	0.003
	Skin	63 (11%)	62 (9%)	1.0 (0.7–1.4)	0.83
	Colorectal	45 (8%)	43 (6%)	0.9 (0.6–1.4)	0.75
	Head and Neck	28 (5%)	37 (6%)	1.4 (0.8–2.4)	0.24
	Gynaecological	23 (4%)	29 (4%)	1.3 (0.7–2.3)	0.43
	Upper GI	15 (3%)	32 (5%)	2.4 (1.2–4.7)	0.008
	Neurological	14 (2%)	16 (2%)	1.3 (0.5–3.5)	0.61
	Lymphohaematopoietic	13 (2%)	29 (4%)	2.3 (1.2–4.5)	0.01
	Ill‐defined and unknown	12 (2%)	17 (3%)	1.6 (0.7–3.7)	0.3
	Other	9 (2%)	10 (1%)	1.1 (0.4–2.8)	0.84

OR, odds ratio; CI, confidence interval; GI, gastrointestinal.

a Statistical test = Chi‐square.

b The significance of prostate treatments was not tested as prevalence was obtained for only tumour groups and not cancer types.

The RTU rate in 2012 in the Remote region was also the lowest of all the regions (Table 2) and the only region to show lower treatment rates after the opening of the WNSWLHD radiotherapy service.

**Table 2:** Radiotherapy utilisation (RTU) rates by residential region

Region	2010			2012			Change % (95% CI)	*P* value^a^
	New RT courses	New cancer cases	RTU rate (%)	New RT courses	New cancer cases	RTU rate (%)		
Bathurst	68	215	32%	81	220	37%	+5% (−4 to 14%)	0.27
Orange	173	577	30%	234	592	40%	+10% (4–15%)	0.0007
Dubbo	154	567	27%	163	577	28%	+1% (−4 to 6%)	0.70
North West	32	102	31%	33	104	32%	+1% (−12 to 13%)	0.90
Remote	26	84	31%	17	84	20%	−9% (−24 to 2%)	0.09

RT, Radiotherapy; RTU, Radiotherapy utilisation; CI, confidence interval.

a Statistical test = Chi‐square.

In the Orange region, the RTU rate increased by 10% over the period, whereas the Dubbo region, which is comparable in terms of population size and medical facilities but primarily serviced by an outreach radiotherapy clinic, increased by 1%.

### Treatment intent

**Figure 3:**
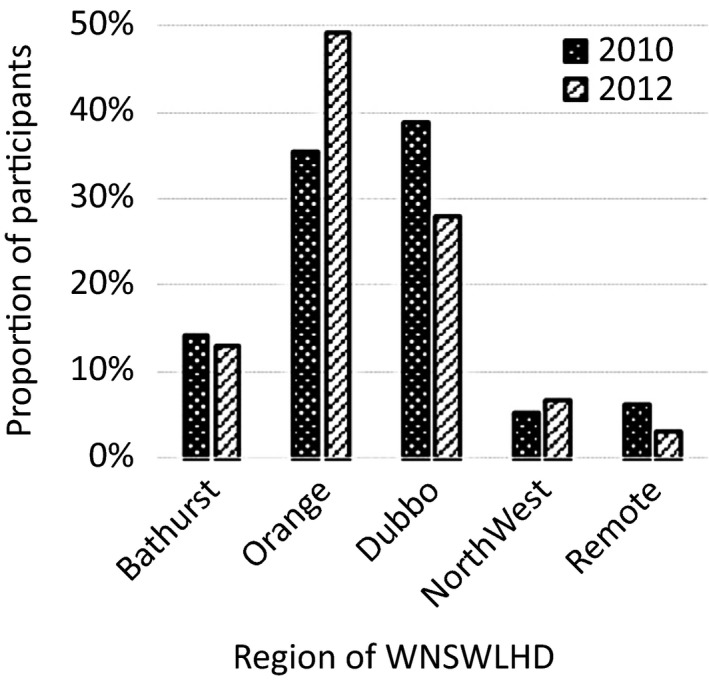
Palliative courses by residential region.

There was no statistically significant difference between the proportion of palliative to curative treatments in 2010 and 2012 (*χ* ^2^(1) = 2.06, *P* = 0.15). By breaking palliative treatments into regional areas, it was found that 78% (62 out of 79) of the additional palliative treatments were for patients from within the Orange region (Fig. 3). The increase in palliative treatments over the 2‐year period in the Orange region was statistically significant (*P* = 0.004). There were no statistically significant increases in treatment rates in the other regions.

### Gender

There were 67 more males treated than females between 2010 and 2012 (Table 1). The proportion of male to female treatments was not statistically different (*P* = 0.086). However, the proportion of male courses to the number of males living with cancer (5‐year prevalence) showed that the increase in treatment rates in 2012 was statistically significant (*P* = 0.002) compared to those treated in 2010. For females, the number of treatment courses to the number living with cancer did not change between 2010 and 2012 (*P* = 0.75).

### Age

There was no statistically significant association between patient age and the presence of a more accessible radiation service in this study.

### Cancer type

Overall, there was minimal change in the type of cancers treated over the study period. The five most common cancer types treated were; breast, prostate, lung, skin and rectum.

Respiratory, upper gastrointestinal (GI) and lymphohaematopoietic tumour groups had the most significant increases in 2012 compared to 2010 with *P*‐values of 0.003, 0.008 and 0.01 respectively. Upper GI and lymphohaematopoietic were small groups and therefore the result could have been an outcome of chance.

However, there is strong evidence that the increase in respiratory cancers is significant because patient numbers were larger. The difference in the number of patients treated between 2010 and 2012 was 19% (66 vs. 97). Of the 31 additional patients treated, 27 (87%) were palliative courses.

## Discussion

The overall RTU increase of 4.1% from 2010 to 2012 shows that the new service has had a beneficial impact in its first full year of operation. Despite the analytical limitations in calculating the RTU rate, the results in this study are comparable to RTU rates seen other studies.

For example, the most relevant, accurate and recent estimation of RTU currently in the literature is a 2004–2006 study that found the utilisation rate for NSW and ACT was 26%.^4^ Our study found the RTU for 2010 and 2012 was, respectively, 29% and 33%, which verifies the reliability of our results.

Our study also found that the average distance travelled for patients to receive treatment decreased from 339 km to 210 km (difference 129 km, *P* = 0.0001). This is a major improvement, however, 210 km is still a distance the majority of patients would not travel daily (note: curative radiation is usually delivered daily over 4–6 weeks and palliative radiation is usually delivered daily over 5–10 days). Thus, many of the patients in the study would have required accommodation, time away from home and work in order to receive radiotherapy.

The study also found a significant improvement in radiotherapy rates in the Orange region. It was the only region in the study to show significantly higher radiotherapy treatment rates (*P* = 0.001) and 78% of additional palliative treatments were from this region. These findings illustrate that distance to a radiotherapy service influenced treatment uptake.

For those living in the Remote region, treatment rates decreased over the study period. As there were small numbers in this group, the results need to be interpreted with caution. This is because there is considerable variability from year to year in the number of people number diagnosed with cancer and the number requiring radiotherapy. Despite this, it is concerning that there was no improvement over the 2‐year period even though every other region showed an increase in treatment rates.

For those in the furthest and most isolated WNSWLHD regions (North West and Remote), it is not feasible, practical or sustainable to build a closer radiotherapy service,^19^ and thus distance will continue to remain a significant deterrent and barrier to accessing radiotherapy services. These regions tend also to have a higher proportion of people from lower socioeconomic backgrounds.^8^ Even with Isolated Patient Travel and Accommodation Scheme (IPTAAS) subsides, travel costs can be substantial and a real barrier to accessing treatment for some people.^20^, ^21^, ^22^It is recommended that alternative strategies be considered to improve access to care for this group of people. One practical solution is to provide higher subsides for travel and accommodation costs. Similar initiatives implemented in other countries have effectively eliminated variations in access between rural and remote regions.^23^

The difference in the RTU rates between the Dubbo and Orange region is an important finding in this study because it shows the difference in treatment uptake between two similar areas that have different health service models. Dubbo, which is similar to Orange in terms of population size, specialists and services, has continued to operate an outreach clinic from Royal Prince Alfred Hospital (RPAH) due to the limited capacity of Central West Cancer Service.

Between 2010 and 2012, the RTU rate in the Orange region increased by 10% (from 30% to 40%), whereas in the Dubbo region the rate only increased by 1% (from 27% to 28%). This suggests that a local radiotherapy service is more effective in increasing the RTU rate than an outreach clinic.

Since this study was conducted a second linear accelerator has opened at Orange, increasing its capacity to treat those from the Dubbo, North West and Remote regions.^9^ A follow‐up study once the second linear accelerator reaches full capacity would strengthen the results of this study and provide a useful comparison. A cost‐benefit analysis of the Orange radiation centre would also be of benefit for planning future oncology services in WNSWLHD.

The reason for the surge in the number of males treated is not known, however, improved access likely to be part of the answer. The types of cancers males received radiotherapy for during the study period was evenly dispersed through all tumour groupings. Therefore, it is unlikely the increase in the number of males treated is due to external factors, such as the number of prostate‐specific antigen (PSA) tests prescribed that year, and thus the number of prostate cancers diagnosed.

Patients with a respiratory cancer also had a significant increase in radiotherapy treatments over the study period. Results show that 87% of the additional respiratory treatments were palliative. The exact reason why this tumour group increased more than any other is not known. The vast majority of additional palliative courses were from within the Orange region, so the improved access to care may be part of the reason for the higher treatment rate.

### Limitations

One limitation of this study was that patient identifiable details were not collected. This meant that it was not possible to determine whether the same patient was treated at a different radiotherapy centre or treated more than once. This limitation was managed by subtracting a retreatment rate of 26.4% to find the number of new treatments and using prevalent cases instead of prevalent people in the denominator estimations.

Using denominator estimations were also an analytical limitation; it would have been preferable to know the actual number of people living with cancer in the region. This was not possible; however, as NSW Cancer Institute's most recent data was from 2008. Conversely, a strength of the NSW Cancer Institute and ABS data was that it exactly matched the defined demographic and tumour groups of this study.

## Conclusion

The opening of a rural radiotherapy unit in WNSWLHD has improved the overall RTU rates. The region closest to the new service, (Orange) was the only area that had a significantly higher number of patients treated in 2012. This was particularly apparent in the number of additional palliative patients who resided within this region. Males and patients with a respiratory cancer also had significantly more radiotherapy in 2012 than 2010.

Since this study has been conducted, a second linear accelerator at Orange has opened, increasing its capacity to treat those from the Dubbo, North West and Remote regions. A follow‐up study once the second linear accelerator reaches full capacity would strengthen the results of this study and show if RTU rates in the regions beyond Orange remain low.

## Conflict of Interest

The authors declare no conflict of interest.
